# Secondary Mesenteroaxial Gastric Volvulus in an Elderly Female: A Rare Presentation of an Occult Diaphragmatic Hernia

**DOI:** 10.7759/cureus.104489

**Published:** 2026-03-01

**Authors:** Rajahamsa Ravala, Surendran Paramsivam, Manish Marlecha, Muthurenganathan PL

**Affiliations:** 1 General Surgery, Sri Ramachandra Institute of Higher Education and Research, Chennai, IND

**Keywords:** acute abdomen, diaphragmatic hernia, gastric volvulus, gastropexy, mesenteroaxial gastric volvulus

## Abstract

Gastric volvulus is a surgical emergency characterized by rotation of the stomach greater than 180°, potentially leading to ischemia and perforation. Mesenteroaxial volvulus associated with a late-presenting diaphragmatic hernia in adults is an uncommon clinical entity.

A 70-year-old woman with diabetes presented with acute abdominal distension and lactic acidosis. Nasogastric decompression yielded 3 L of coffee-ground aspirate, and computed tomography (CT) identified a mesenteroaxial gastric volvulus. Emergency laparotomy revealed a left-sided diaphragmatic hernia containing the gastric antrum and pylorus. Management included detorsion, reinforcement of a focal ischemic area on the posterior gastric wall, diaphragmatic repair, and gastropexy.

This case highlights the silent nature of congenital diaphragmatic defects in the elderly, which can present as life-threatening acute scenarios. It emphasizes the importance of immediate and adequate resuscitative measures, coupled with early diagnosis and definitive treatment.

## Introduction

Gastric volvulus, derived from the Latin volvere meaning “to roll,” is a rare but life-threatening condition in which the stomach rotates more than 180° around its axis. This torsion can result in closed-loop obstruction, vascular compromise, and eventual perforation. Although the incidence peaks after the fifth decade of life, the condition remains uncommon. Anatomically, gastric volvulus is classified into organoaxial and mesenteroaxial types. Organoaxial rotation occurs along the cardiopyloric axis, whereas mesenteroaxial rotation occurs perpendicular to this axis, causing the stomach to fold upon itself with superior displacement of the antrum and pylorus [1,2].

Etiologically, gastric volvulus is categorized as primary, arising from ligamentous laxity, or secondary, resulting from anatomical defects such as paraesophageal or diaphragmatic hernias [2,3]. While diaphragmatic hernias are often detected in childhood, they may rarely remain latent until adulthood, presenting acutely with complications such as gastric volvulus. The classical clinical presentation is Borchardt’s triad, which includes acute epigastric pain, intractable retching without emesis, and inability to pass a nasogastric tube [1]. While plain radiography may suggest the diagnosis, multidetector computed tomography (CT) has become the gold standard for identifying the axis of rotation and assessing associated complications [3]. Given reported mortality rates of 30%-50% in acute cases, prompt surgical intervention remains the cornerstone of management [4].

## Case presentation

A 70-year-old woman with diabetes presented with a one-day history of acute abdominal pain and progressive distension. Physical examination revealed a grossly distended abdomen and absent bowel sounds. Initial laboratory evaluation demonstrated anemia and significant lactic acidosis (Table 1).

**Table 1:** Lab parameters on arrival showing anemia and marked lactic acidosis.

Parameter	Patient Value	Normal Ranges
Hemoglobin (Hb)	7.7 g/dL	12-15 g/dL
Total Counts	12,090 /mm^3^	4,000-10,000 /mm^3^
Platelets	4.1 L/mm^3^	1.5-4.5 L/mm^3^
pH	7.07	7.35-7.45
HCO_3_	16 mmol/L	22-26 mmol/L
Lactate	14.2	0.2-1.8

**Figure 1:**
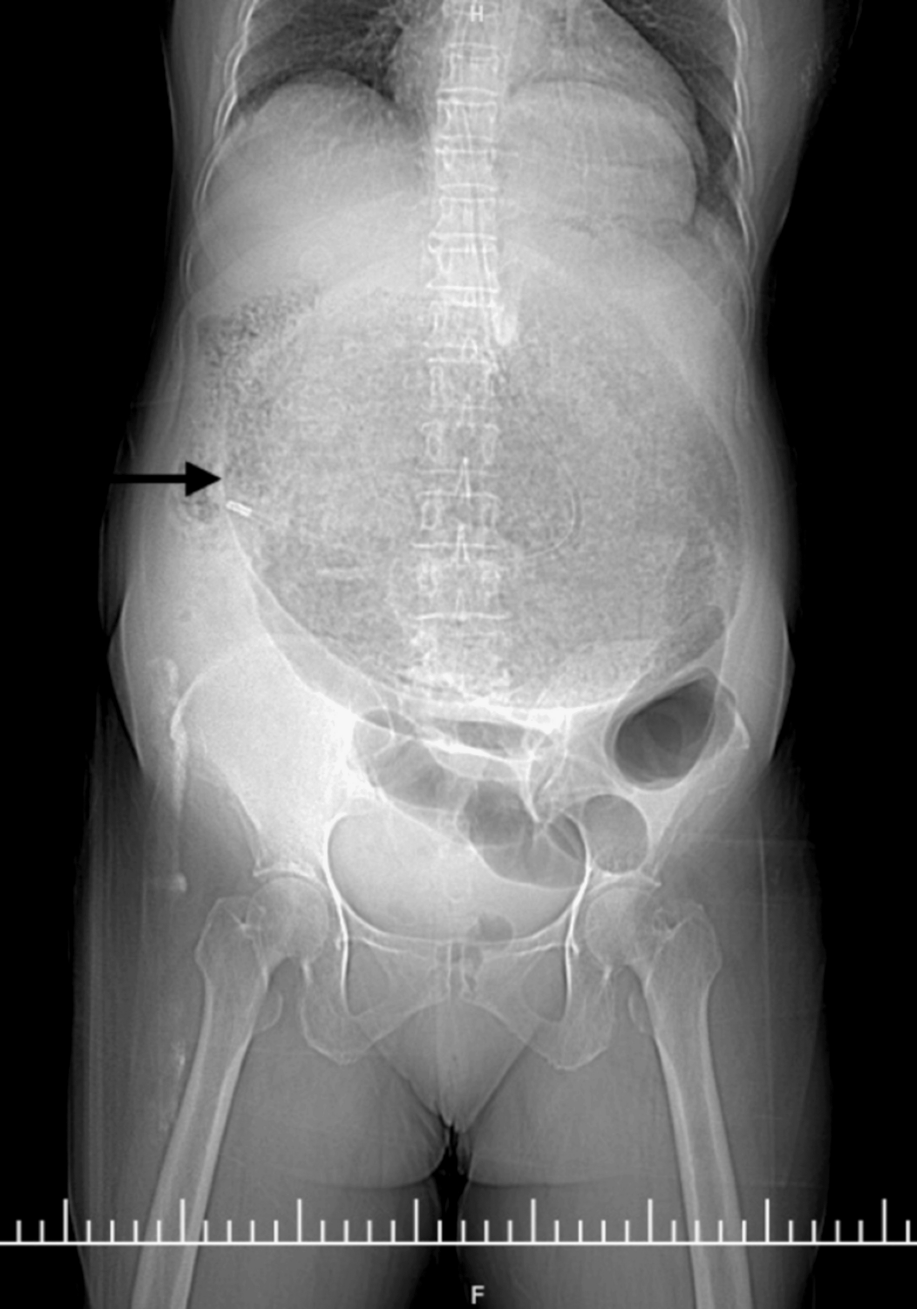
Computed tomography (CT) scout image showing a grossly distended stomach with a nasogastric tube in situ (arrow).

**Figure 2:**
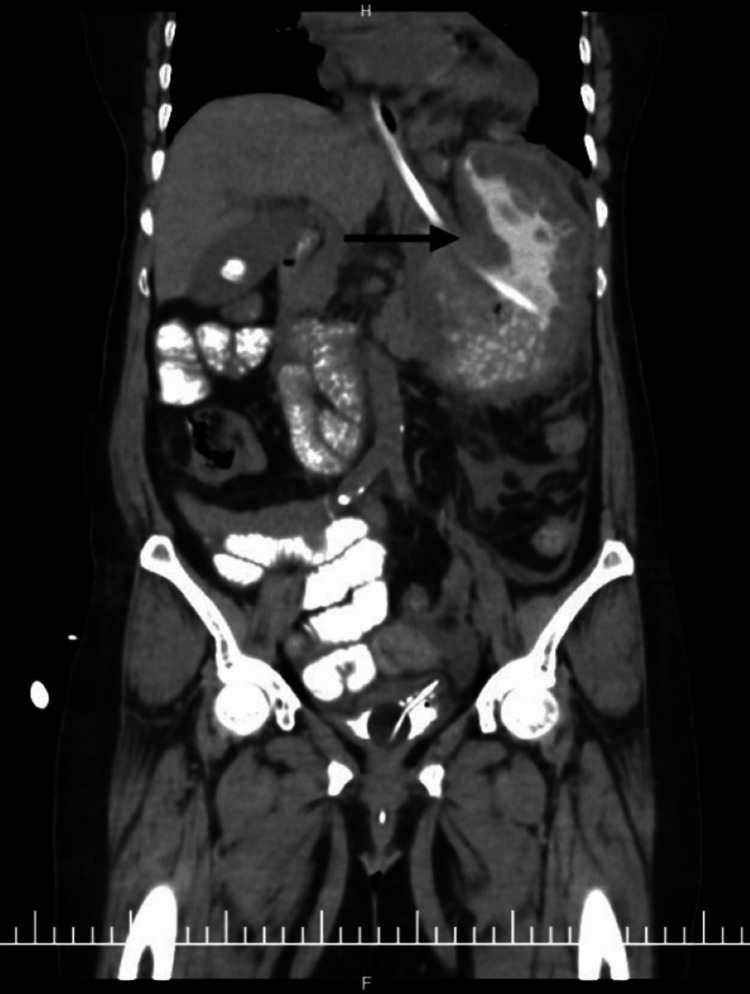
Coronal view of contrast-enhanced computed tomography (CECT) of the abdomen showing rotation of the stomach (arrow) with Ryle’s tube in situ.

**Figure 3:**
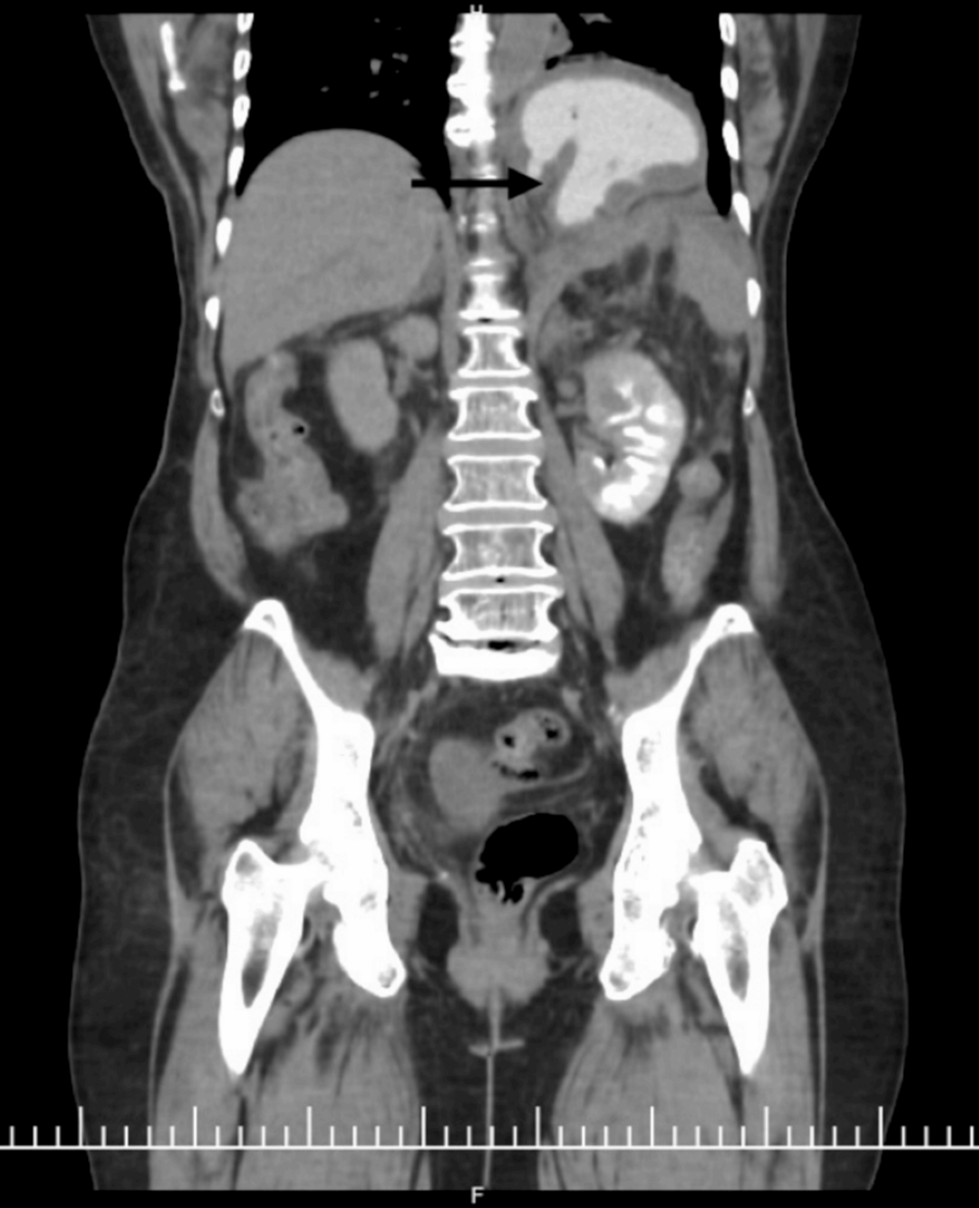
Coronal view of contrast-enhanced computed tomography (CECT) of the abdomen with the pylorus displaced superiorly above the fundus (arrow).

**Figure 4:**
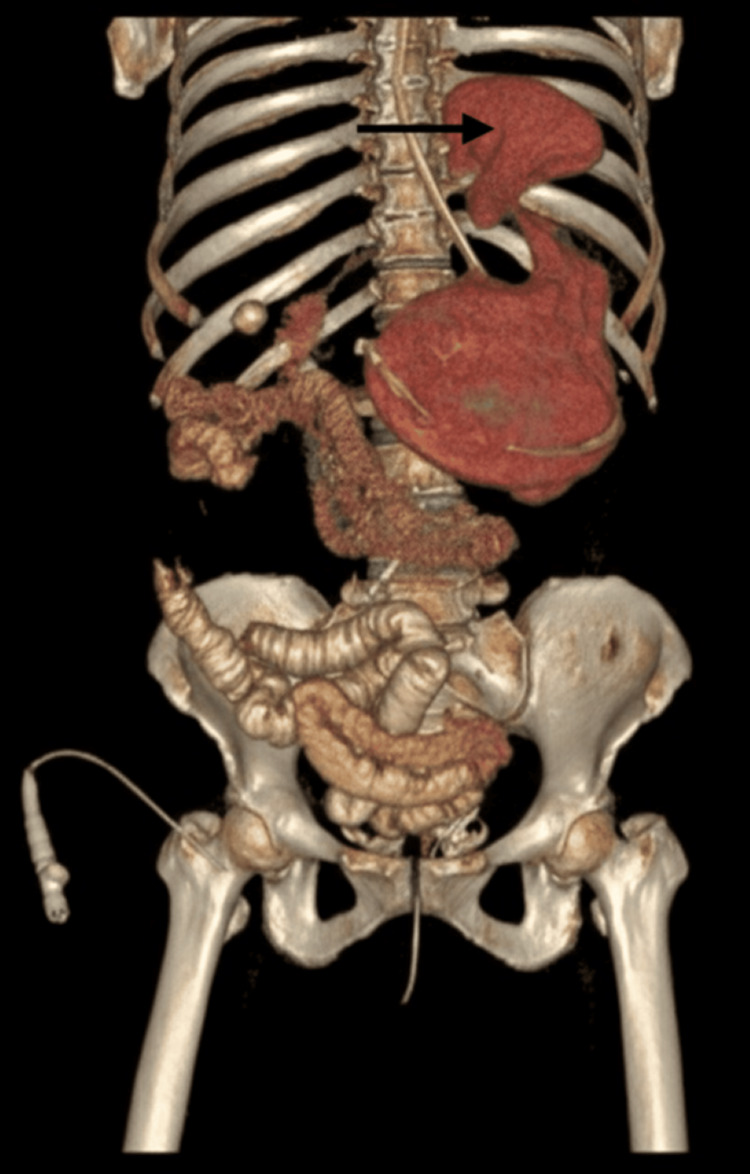
Three-dimensional reconstructed computed tomography (CT) image of the abdomen demonstrating the mesenteroaxial gastric volvulus (arrow).

A nasogastric tube was placed with difficulty, resulting in immediate evacuation of 3 L of coffee-ground material. The patient subsequently developed hemodynamic instability, requiring inotropic support and blood transfusion. Initial CT scout imaging demonstrated marked gastric distention (Figure 1). Contrast-enhanced computed imaging (CECT) revealed a mesenteroaxial gastric volvulus (Figure 2), with the antrum and pylorus situated superior to the fundus (Figure 3) without evidence of pneumoperitoneum, and a large calculus within the gallbladder. Three-dimensional (3D) volume-rendered reconstruction further delineated the abnormal gastric rotation (Figure 4). 

Following stabilization, an exploratory laparotomy was performed. Intraoperatively, a grossly hypertrophied stomach was found folded transversely. The pylorus and supracolic omentum were incarcerated within a large, left-sided diaphragmatic hernia sac. After adhesiolysis and detorsion, the stomach was reduced, and the supracolic omentum was excised. A focal area of suspicious discoloration was noted on the posterior gastric wall. Given the localized nature of the insult, this was managed with seromuscular reinforcement using 2-0 Vicryl sutures.

The diaphragmatic defect was repaired primarily, and mesh was deferred due to the presence of reactive fluid and the patient’s glycemic status. An anterior gastropexy was performed to prevent recurrence. A concomitant cholecystectomy was performed for the incidental finding of cholelithiasis. The postoperative course was uneventful, with resumption of a solid diet by postoperative day 3 and discharge on day 7. At one-month follow-up, the patient remained asymptomatic.

## Discussion

The presentation of mesenteroaxial gastric volvulus in a 70-year-old patient is a rare clinical event, particularly when secondary to an occult congenital diaphragmatic hernia. This case is distinguished by the rarity of the presentation, massive gastric sequestration, and rapid physiological recovery following timely surgical intervention.

The Carter modification of the von Haberer classification remains the clinical standard for identifying the axis of torsion in gastric volvulus [1]. While organoaxial volvulus accounts for the majority of adult cases, mesenteroaxial rotation is more commonly observed in pediatric populations and is rare in adults [2]. When it does occur in adults, it is almost exclusively secondary to an underlying anatomical lead point, most commonly a diaphragmatic or paraesophageal hernia [3]. In the present case, the left-sided diaphragmatic hernia provided sufficient space for superior migration of the antrum and pylorus. It is hypothesised that the defect was partially occluded by intra-abdominal viscera or omentum until age-related loss of tissue elasticity precipitated the gastric volvulus. The presence of a hypertrophied stomach suggests a chronic-on-acute process, wherein intermittent subclinical torsion may have occurred over several years, culminating in acute incarceration due to progressive adhesions within the hernia sac [2]. 

Management of gastric wall viability during volvulus requires nuanced surgical judgment. Owing to the stomach’s rich collateral blood supply, a conservative approach is often feasible when ischemic changes are localized [4]. In this case, focal posterior wall discoloration was managed with seromuscular reinforcement rather than formal gastric resection, minimizing operative morbidity in a hemodynamically fragile patient.

The decision to perform primary diaphragmatic repair without mesh was deliberate. In emergency settings, particularly in the presence of reactive fluid and uncontrolled diabetes, the risk of prosthetic infection outweighs the potential benefit of mesh reinforcement [3]. Definitive fixation via gastropexy remains essential in cases of secondary gastric volvulus. Anchoring the stomach to the anterior abdominal wall significantly reduces the risk of recurrence, even in the presence of age-related ligamentous laxity [1,2].

## Conclusions

Gastric volvulus should be considered in elderly patients presenting with acute abdominal distension, especially when accompanied by unexplained lactic acidosis. Awareness of Borchardt’s triad and early use of CT can facilitate prompt diagnosis and timely surgical intervention. Early operative management allows correction of the volvulus, improvement in physiological parameters, and prevention of progression to gastric ischemia or perforation. This case highlights the importance of early recognition and decisive management in achieving a favorable outcome in an otherwise uncommon but potentially life-threatening condition.
